# Long term follow-up of a simplified and less burdened pancreatic duct ligation model of exocrine pancreatic insufficiency in Goettingen Minipigs

**DOI:** 10.1186/s12876-020-01541-3

**Published:** 2020-11-30

**Authors:** Andreas Minh Luu, Alexander Brock, Sabrina Ritz, Sandra Junghänel, Ingo Aldag, Stella Edskes, Marcus Hartmann, Michael Hessler, Michael Praktiknjo, Philip Arnemann, Christian Ertmer, Waldemar Uhl, Juergen Schnekenburger, Torsten Herzog

**Affiliations:** 1 Department of General Surgery, St. Josef Hospital, Ruhr University Bochum, Gudrunstrasse 56, 44791 Bochum, Germany; 2 Biomedizinisches Technologiezentrum, Westfaelische Wilhelms-Universitaet Muenster, Mendelstrasse 17, 48149 Muenster, Germany; 3 Cilian AG, Johann Krane Weg 42, 48149 Muenster, Germany; 4 Klinik Für Anaesthesiologie, Operative Intensivmedizin Und Schmerztherapie, Universitaetsklinikum Muenster, Albert Schweitzer Campus 1, Gebaeude A1, 48149 Muenster, Deutschland; 5 Department of Internal Medicine, University of Bonn, Venusberg-Campus 1, 53127 Bonn, Germany

**Keywords:** Exocrine pancreatic insufficiency, Goettingen minipig, Pancreas, Pancreatic duct ligation, Pancreatic enzyme replacement therapy

## Abstract

**Background:**

Pancreatic duct ligation in a minipig model leads to exocrine pancreatic insufficiency (EPI). This allows the study of digestive processes and pancreatic enzyme replacement therapies. However, detailed descriptions of the surgical procedure, perioperative management, a determination of exocrine pancreatic insufficiency are scarce in the literature. Data of the long-term health status of minipigs upon EPI induction are still not available. Therefore, the present study describes in detail an experimental approach to the induction of exocrine pancreatic insufficiency via pancreatic duct ligation in minipigs and the long term follow up of the animal’s health state.

**Methods:**

14 Goettingen minipigs underwent pancreatic duct ligation via midline laparotomy for the induction of exocrine pancreatic insufficiency. Fecal fat content, fat absorption, chymotrypsin levels, body weight and blood vitamin and glucose levels were determined.

**Results:**

Exocrine pancreatic insufficiency was successfully induced in 12 Goettingen minipigs. Two minipigs failed to develop exocrine insufficiency most likely due to undetected accessory pancreatic ducts. All animals tolerated the procedure very well and gained weight within 8 weeks after surgery without requiring pancreatic enzyme replacement therapy. The follow up for approx. 180 weeks showed a stable body weight and health state of the animals with normal blood glucose levels (Table 1). From approx. 130 weeks post pancreatic duct ligation, all animals were supplemented with pancreatic enzymes and vitamins resulting in blood concentrations almost within the reference range.

**Conclusions:**

Pancreatic duct ligation in minipigs is an excellent method of inducing exocrine pancreatic insufficiency. It is important to identify and ligate accessory pancreatic ducts since persistence of accessory ducts will lead to maintenance of exocrine pancreatic function. The EPI model caused no persistent side effects in the animals and has the potential to be used in long-term EPI studies with up to 100 weeks post-OP without supplementation with enzymes and vitamins.

## Background

Exocrine pancreatic insufficiency (EPI) leads to maldigestion of food and malabsorption of nutrients due to an impaired secretion or activity of pancreatic digestive enzymes. EPI can be induced by several disorders like cystic fibrosis (CF), acute (AP) or chronic pancreatitis (CP), cancer or diabetes mellitus or as a complication of gastrointestinal surgery [1–3]. Progression, severity and occurring comorbidities in the course of EPI varies strongly between patients and correlates with the etiology of preliminary diseases and the lifestyle of the affected person (e.g. nutrition, smoking, alcohol consumption etc.). For patients affected by exocrine pancreatic insufficiency (EPI) symptoms like abdominal pain, diarrhea or flatulence are everyday struggles accompanied by fatty, lose stool (steatorrhea), weight loss and malnutrition [3, 4]. The symptoms significantly decrease the quality of life and malnutrition especially of fat soluble vitamins (vitamins E, D, K and A) subsequently results in other severe diseases like osteoporosis, osteopenia or low trauma fracture [5, 6].

The development and testing of EPI treatments requires suitable in vivo models of the complex disease. The ligation of the pancreatic duct in minipigs is a well-established model to investigate the influence of EPI on cleavage and absorption of food [1]. Because of the high similarity in digestion compared to humans and the feasibility to ligate the separated pancreatic duct, pigs and especially minipigs are preferred as EPI model to study digestion and absorption of metabolites [7–15]. Ileo-cecal fistulated EPI minipigs were used for the analysis of digestive processes [7]. Also, the effect of a pancreatic enzyme replacement therapy (PERT) was analyzed in EPI pigs and an improvement of this type of therapy was reported [8]. The selection of the suitable EPI minipig model depends on the required data sets. Fistulated EPI minipigs allow [8] the analysis of digestive enzyme mechanisms inside the intestine. The disadvantages of the model are the high demands on the housing and animal care condition, possible infections and the requirements on the rather liquid feed consistency. Pancreatic duct ligation without fistulation is sufficient for a streamlined PERT study and allows keeping in groups during non-study periods and comes closer to the 3-R principle.

The surgical procedure of pancreatic duct ligation leads to a complete loss of pancreatic enzyme secretion resulting in malnutrition [9–14]. The successful surgery of minipigs needs training and knowledge of the porcine gastrointestinal tract. Accurate anatomical and physiological knowledge regarding relevant differences between pigs and humans is crucial prior to surgery. In contrast to humans, the bile duct and pancreatic duct in pigs drain into the duodenum separately [15, 16]. Therefore, ligature of the pancreatic duct does not result in extrahepatic cholestasis. In general, the pancreatic duct provides an extra parenchymal segment between the pancreatic head and the duodenum [17]. Occlusion of this segment allows technically feasible induction of exocrine pancreatic insufficiency.

The available literature lacks a clear and detailed description of the surgical approach. The effects of the surgery and postoperative determination of exocrine pancreatic insufficiency are not reported in studies using EPI minipigs models [15, 18–23]. Moreover, the intended long-term use of EPI minipigs may raise issues regarding animal health and nutrition status. EPI may affect body weight and the general health state, blood glucose levels and the absorption of fat-soluble vitamins. Digestive enzyme depleted minipigs require supply of fat soluble vitamins and digestive enzymes [24]. This effect of malnutrition is close to the typical course of disease in human patients [25, 26]. Therefore, EPI minipigs require a strategy of supplementation of digestive enzymes and the compensation of EPI dependent malnutrition.

The aim of this work is to provide a detailed description of the surgical approach to pancreatic duct ligation, the perioperative management, and the determination of exocrine pancreatic insufficiency in minipigs. Furthermore, we describe for the first time the long-term follow up of the EPI animals and the postoperative diet requirements, food supplements and treatments, which are essential for the optimal health state of the non-fistulated EPI minipigs.

## Methods

### Animals

Pancreatic duct ligation was performed in 14 female Goettingen minipigs which were obtained from Ellegaard Goettingen Minipigs A/S, Dalmose, Denmark. Genders were not mixed to avoid gender-specific differences such as size and physique and the associated different metabolism of the animals and thus to increase the comparability of the experiments. Furthermore, in order to comply with the 3-R principle and the national animal welfare regulations, sham operated animals were omitted and only animals that could be used to study digestion or the effectiveness of PERT after induction of the EPI model of pancreatic duct ligation were allowed. Since the animals were used as their respective controls on high fat diet, no further control animals for PERT studies were required. Prior to surgery, the mean age was 21.9 (± 3.7) months and the mean weight was 34.6 (± 3.2) kg. The pigs were kept in groups of 2–4 animals and fed twice a day. The stables are modular variable by easily installable metal mesh, the box size can be changed during the experimental series, to keep animals separate in single boxes. For long-term usage of this EPI model resting phases of at least 6 weeks between the experimental series were included.

All animal experiments were approved by the ethics committee of the University of Munster and conducted according the guidelines of the animal use and care committee of the University of Muenster and the North Rhine Westphalia State Agency for Nature, Environment, and Consumer Protection (LANUV) (no. 84-02.04.2015.A021/01).

The experiments further complied with the ARRIVE guidelines and were carried out in accordance with the National Institutes of Health Guide for the Care and Use of Laboratory Animals.

### Preoperative management and anesthesia

After a 24 h fasting period prior to surgery, the pigs were sedated with racemic ketamine (15 mg/kg intramuscular (IM); Vetoquinol, Ravensburg, Germany) and azaperon (2 mg/kg IM; Elanco, Bad Homburg, Germany). Anesthesia was induced with propofol (3 mg/kg intravenous (IV); Fresenius Kabi, Bad Homburg, Germany). Anesthesia was maintained using a Draeger Cato® closed circuit respiratory flow system (Draegerwerk AG & Co. KgaA, Luebeck, Germany) and an endotracheal tube with 1.8–2.0% endtidal isofluran (Abbvie, Chicago, IL, USA). Analgesia was provided by administration of butorphanol (0.3 mg/kg IV; Vetoquinol, Ravensburg, Germany). Muscle relaxation was not necessary. Preoperative antibiotic prophylaxis was achieved with enrofloxacin (2.5 mg/kg IM; Bayer, Leverkusen, Germany).

### Surgery for pancreatic duct ligation

**Fig. 1:**
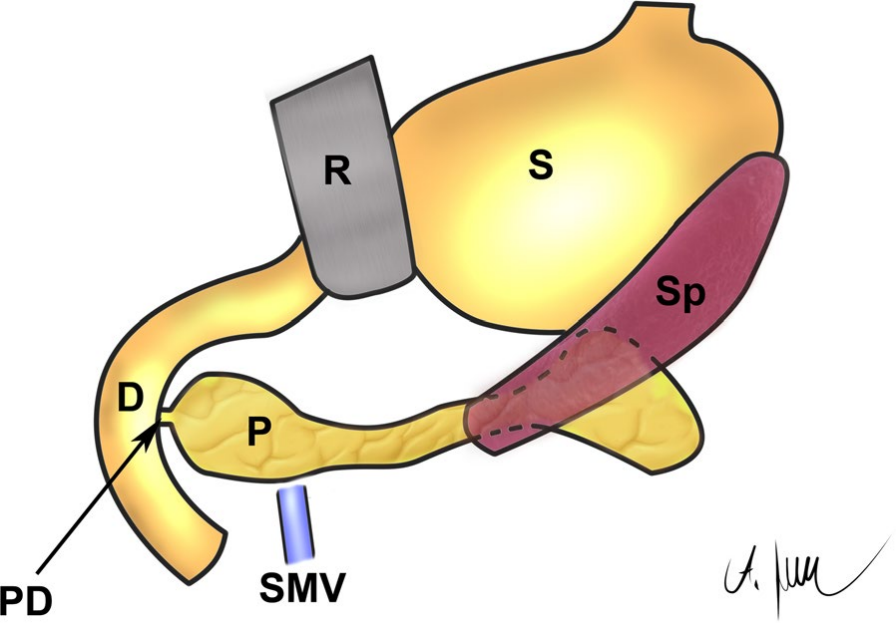
Schematic drawing of the upper abdomen of a Goettingen minipig. P = pancreas, PD = pancreatic duct, CBD = common bile duct draining into the duodenum separately, SP = spleen, S = stomach, R = retractor, D = duodenum, SMV = superior mesenteric vein. Thus drawing was created by AML

**Fig. 2:**
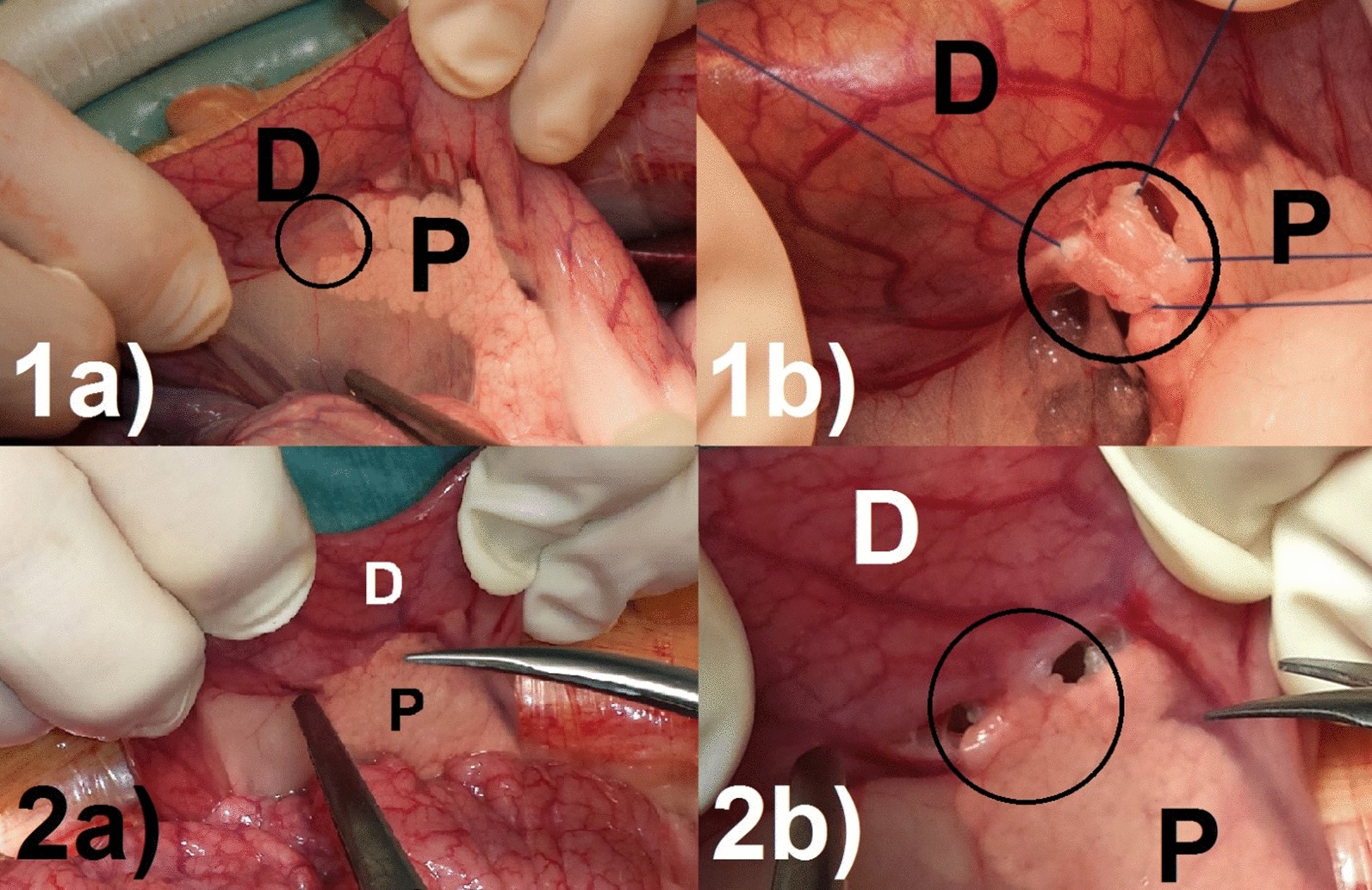
Intraoperative view of the pancreatic duct (encircled) embedded in a membrane between pancreas (P) and duodenum (D) prior to (1a, 2a) and after (1b, 2b) separation. The pancreatic duct is difficult to identify on image 2a

Midline laparotomy was performed by default. The incision length was approximately 15 cm beginning 2 cm below the xiphoid process. An Alexis® wound retractor provided sufficient exposure of the upper abdomen (Applied Medical, Rancho Santa Margarita, CA, USA). A schematic drawing of the upper abdomen is shown in Fig. 1. In contrast to humans, the porcine spleen is larger and not located dorsolaterally [27]. The typical location was the left upper abdominal quadrant extending to the epigastrium. The spleen was carefully held aside to avoid organ- or vessel rupture. Small bowel loops were also held aside which provided access to the pancreaticoduodenal area. The extraparenchymal segment of the pancreatic duct was always embedded in a membrane between the duodenum and the pancreatic head (Figs. 1, 2). Preparation was performed carefully because this segment of the pancreatic duct could be very short and difficult to identify (Fig. 2a). A missed accessory pancreatic duct could lead to failure of induction of exocrine pancreatic insufficiency. Once the pancreatic duct was identified and isolated, it was ligated with a 4–0 non-absorbable polypropylene suture as shown in Fig. 2b (Prolene®, Johnson & Johnson Medical GmbH, Norderstedt, Germany). Subsequently, the duct was cut between two ligatures, leaving the closed ends in the membrane between pancreas and duodenum. The common bile duct was never encountered due to separate drainage into the duodenum. In a next step the abdominal wall was closed. Peritoneum and fascia were closed using a continuous suturing technique with an absorbable 3–0 suture (Prolene®, Johnson & Johnson Medical GmbH, Norderstedt, Germany). The skin was closed with an absorbable intracutaneous 4–0 suture to avoid manipulation of the wound by the pigs (Maxon®, Covidien, Medtronic, Meerbusch, Germany). Spray-on plaster (Spray plaster®, Hansaplast, Hamburg) completed the wound closure. Application of a dressing was not necessary.

### Postoperative management

Antibiotic treatment with enrofloxacin (2.5 mg/kg/day IM; Bayer, Leverkusen, Germany) was maintained five days postoperatively. Analgesia was provided by administration of butorphanol (0.3 mg/kg IM; Vetoquinol, Ravensburg, Germany) twice and carprofen (4.0 mg/kg IM; Pfizer, New York, NY, USA) once a day.

### Dietary

Animals were fed twice a day with 300 g pig feed (Minipig Haltung Altromin 9021, Lage, Germany) at a daily energy intake of 1,700 kcal/ 7,093 kJ.

In three experimental series, one before and two six weeks after pancreatic duct ligation, the pigs were given a high fat diet (composition 1) twice a day for one week consisting of 200 g pig feed, 75 g cream (30% fat), and 25 g olive oil (1,968 kcal/8,236 kJ). During the third experimental series pigs were supplemented with approx. 130.000 U of a commercial pancreatin preparation (2063 U pancreatic lipase/g fat).

Comparable PERT approaches were conducted from 96–126 weeks post OP in several studies. Among others pigs were not supplemented (placebo) or supplemented with again approx. 130.000 U pancreatic lipase fed with two different high fat dietary compositions (2: low fibre consisting of 200 g pig feed (Minipig Spezial Altromin 902,014, Lage, Germany), 75 g cream (30% fat), and 25 g olive oil (2,474 kcal/10,340 kJ) and 3: high fibre consisting of 200 g pig feed (Minipig Haltung Altromin 9051, Lage, Germany), 83 g cream (30% fat), and 28 g olive oil (1,937 kcal/8,112 kJ)).

To stop the increasing weight gain in long-term husbandry, the fodder was changed to 200 g twice a day of Altromin® 9051 after approx. 120 weeks post-OP so that the daily intake was 926 kcal/ 3,864 kJ. In addition, approx. 137 weeks post-OP the animals were given twice a day 2 g of pancreatic enzymes (pancreatic lipase activity 50,000 U/g) to the fodder and were supplemented weekly with 5 g Konivet Multi (vitamins A, D3, E, C, B1, B2, B6, B12, K3, nicotinamide, calcium-D-panthotenat, folic acid) und 1 g Konivet E250 (Konivet GmbH, Essen, Germany). Additionally, vitamin B12 (2000 µg Cyanocobalamin, Jenapharm, Mibe GmbH, Brehna, Germany) was injected intramuscularly every four months.

### Sample collection and preparation

Experimental series before and after pancreatic duct ligation were conducted for 7 days. Animals were weighed prior to starting the diet and on day 7. Feces were collected on days 5, 6, and 7 three times a day at 8 a.m., 1 p.m., and 6 p.m. They were weighed and homogenized in a mixer (Rotor Gastronom GK-900®, Feuma Gastromaschinen, Goessnitz, Germany), and samples for chymotrypsin activity determination were taken. For determination of dry mass, fat content, and acid-insoluble ash, feces were dried in aliquots of approximately 20 g feces in 50 ml tubes at 60 °C and 10 mbar. Fasting blood glucose was measured with a glucometer (Contour XT®, Bayer, Leverkusen, Germany) before surgery and 6 weeks after by ear vein puncture. The animals fasted 16 h prior determination.

### Determination of fat content and absorption

The fat content of the feces and diet was determined by the modified method by Berstadt et al. based on the method of van de Kamer et al. [28, 29]. Briefly, 10 ml 33% KOH (potassium hydroxid) solution and 40 ml ethanol with 0.4% isoamylalcohol were added to 2–3 g feces. The sample was then boiled for 20 min. After cooling down, 25% HCl (hydrochloric acid) solution was added followed by 50 ml petroleum ether. After 1 min of shaking, the organic phase was transferred to a new vessel and the ether removed via distillation. The remaining components were dissolved in 20 ml ethanol, and the sample was titrated with.

## M NaOH (sodium hydroxid) in a titrator (Titrando 902®, Metrohm GmbH & Co. KG, Filderstadt, Germany) to a pH of 9.5. The fat content was determined using Eq. 1.

Equation 1 for calculating fecal fat content [21].

1 $$fat\;content\;(\% ) = \frac{{V\left( {NaOH} \right)}}{m\left( g \right)\;sample}*11.814$$

To calculate the coefficient of fat absorption (CFA), it was required to determine the concentration of acid-insoluble ash in the diet and feces.

Acid-insoluble ash in the feces and diet served as an indicator following the procedure of Van Keulen and Young [30]. 3–4 g dried feces/diet were burned in a pan at 600 °C. The ash was suspended in 37.5% HCl, heated to boiling point, and filtrated with ashless filter paper. The filter was washed with hot distilled water and again burned as described above. After cooling down, the acid-insoluble ash content was determined gravimetrically.

The CFA was determined using Eq. 2.

Equation 2 for calculating the coefficient of fat absorption (CFA) [27].

2 $$ \begin{aligned}   cfa(\% ) &  = 100 - \frac{{acid\;insoluble\;ash\;in\;diet\;(\% )}}{{acid\;insoluble\;ash\;in\;feces\;(\% )}} \\     & *\frac{{fat\;in\;feces}}{{fat\;in\;diet}}*100 \\  \end{aligned}  $$

### Determination of chymotrypsin activity

Chymotrypsin activity was determined with the Chymotrypsin Activity Kit by Immundiagnostik AG, Bensheim, Germany (K 6990). Briefly, aliquots of 120 mg feces were extracted in solvents buffer (Immundiagnostik, K 6990 SOL) with the fecal sample preparation kit (Roche No. 10745 804, Mannheim, Germany,). 100 µl of chymotrypsin containing solvent buffer was added to 2 ml of substrate solution in a 3 ml cuvette at 30 °C. Absorption was measured after 1, 2, and 3 min at a wavelength of 405 nm with a Biochrom® Libra S12 Spectrophotometer (Biochrom, Cambridge UK). Chymotrypsin activities were calculated using Eq. 3.

Equation 3 for determining fecal chymotrypsin activity (Immundiagnostik AG, Bensheim, Germany).

3 $$U/g\;stool\; = 212 \times \Delta E_{405} /\min .$$

### Determination of total fat absortion

Calculation of total fat absorption is done using Eq. 4

4 $$Calc. total fat absorption \left[g\right]=cfa\frac{\left(\%\right)}{100}*dietary fat amount [g]$$

### Health monitoring

To monitor the health status of every animal, score sheets were used covering body weight, general state of health, spontaneous behavior and medical findings. The degree of stress was counted for each animal using 0 = no stress, 1 = light stress, 2 = medium stress, up to 3 = maximum stress followed by immediate experimental break and euthanasia of the animal. Among others, pigs were weighed approx. every other week in the 180 weeks post-OP follow up phase.

During the first approx. 120–137 weeks post-surgery, the animals received only maintenance feed and were not supplemented. Subsequently, the supplementation of the animals was started. The blood chemistry was analyzed before and after supplementation of pancreatic enzymes and vitamins. Therefore, blood samples were taken by ear vein puncture under a mild sedation with Stresnil (2 mg/ kg bodyweight, Elanco Deutschland GmbH, Bad Homburg, Germany) and Ketamin (15 mg/kg bodyweight, Wirtschaftsgenossenschaft deutscher Tierärzte eG, Garbsen, Germany) by intramuscular injection. The blood samples were analyzed by the laboratory Laboklin GmbH (Bad Kissingen, Germany).

### Euthanasia

For euthanasia, premedication was given by intramuscular injection of ketamine (15 mg/kg body weight) and xylazine (2 mg/kg body weight). After onset of effect the sedation was deepened by inhalation anesthesia with 2–4% isoflurane in 10 L/min room air or 2 L/min oxygen, which was applied via an anaesthetic mask. Finally, T61 (Embutramid: 0.1–0.3 mL/kg body weight) was injected intracardially.

### Statistical analysis

All data were expressed as mean (± standard deviation (SD). Body mass, fecal fat content, CFA, and blood glucose were compared using a paired one-tailed t-test. SPSS® 24 (IBM, Armonk, NY, USA) was used for statistical analysis.

## Results

The animals were delivered by a specialized breeder and allowed to acclimate for at least 8 weeks. The animals then underwent surgery for pancreatic duct ligation, followed by an 8 weeks recovery period. The successful establishment of EPI was tested by determination of chymotrypsin activity in feces. Animals with an EPI were then used for repeated PERT studies and the animal health state and dietary and nutrient requirements documented for the following years.

Pancreatic duct ligation (PDL) was performed successfully (Figs. 1, 2) and without postoperative complications in all cases. The scoring of the animals was postoperative day 4–5 set to 2. All minipigs showed normal mobility and behavior within 12 h of surgery, although a relieving posture was maintained for 3–4 days. After 7 days feed intake, behavior and physical condition returned to normal in all animals. The animals were attentive, showed no signs of pain and inflammation and a normal feeding behavior. Weight was maintained after surgery while following a regular pig diet (Table 1).

**Table 1:** Clinical parameters

	before PDL	after PDL
Fasting blood glucose levels	68.9 (± 23.5) mg/dl	54.8 (± 8.1) mg/dl
Body weight	34.6 (± 3.2) kg	35.4 (± 3.4) kg
Fat content of feces	9.02 (± 2.44) %	30.89 (± 4.25) %
Coefficient of fat absorption	90.01 (± 2.75) %	24.21 (± 8.34) %

Mean with standard deviation of fasting blood glucose levels and body weight before and 6 weeks after pancreatic duct ligation (PDL). In addition, fecal fat content and coefficient of fat absorption (CFA) were determined after feeding a high-fat diet for one week

No enzyme supplementation was given at this time. All animal health care scores were within the normal range.

**Fig. 3:**
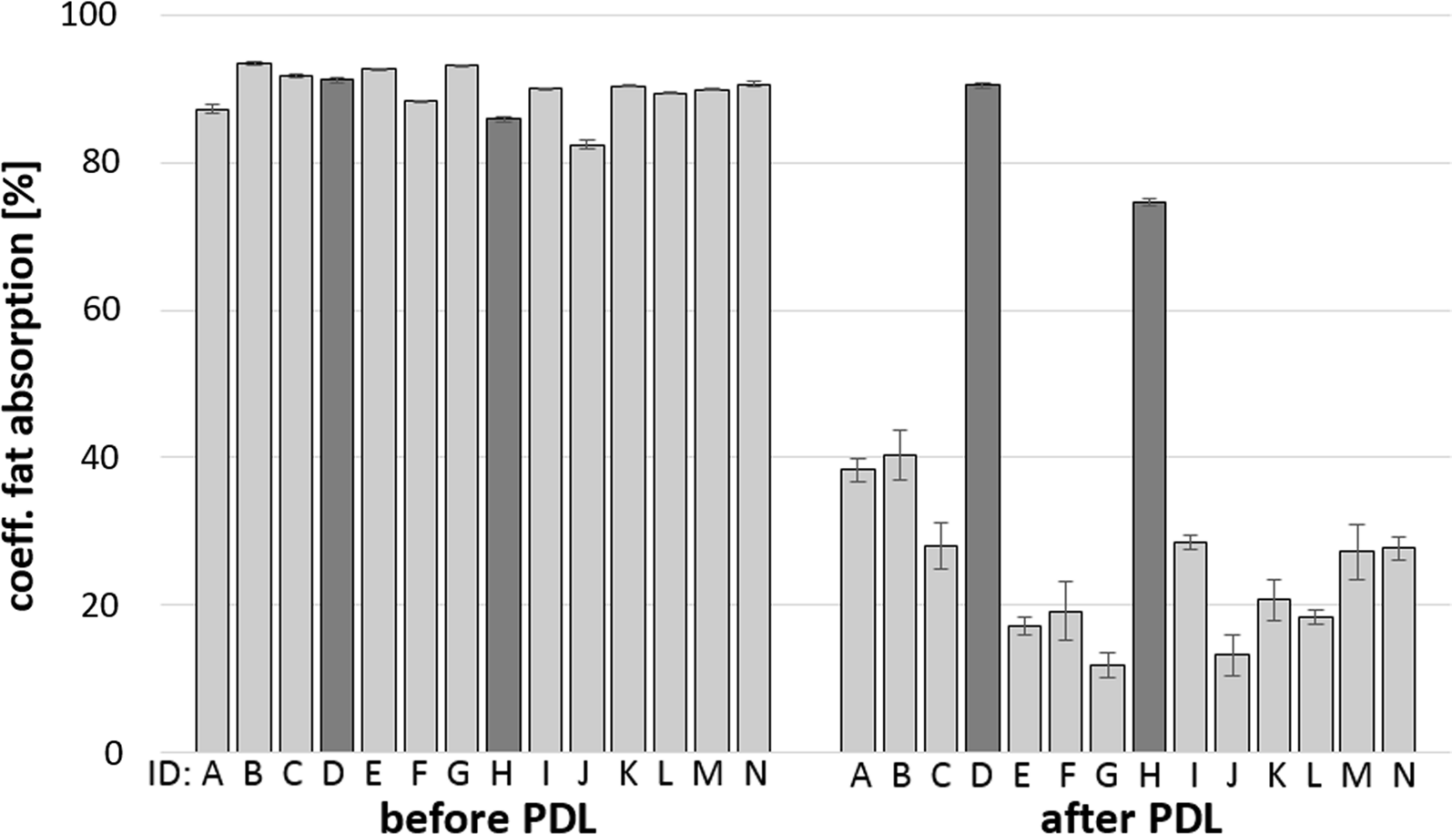
Coefficient of fat absorption (CFA) in minipigs before and six weeks after pancreatic duct ligation (PDL). Pigs were fed with a high-fat diet (18.5%) for seven days twice a day. Feces were collected on days 5–7, and fat absorption was then determined. Animal identification (ID) ranged from A to N. Dark columns indicate animals D and H with only a minor reduction in fat absorption

**Fig. 4:**
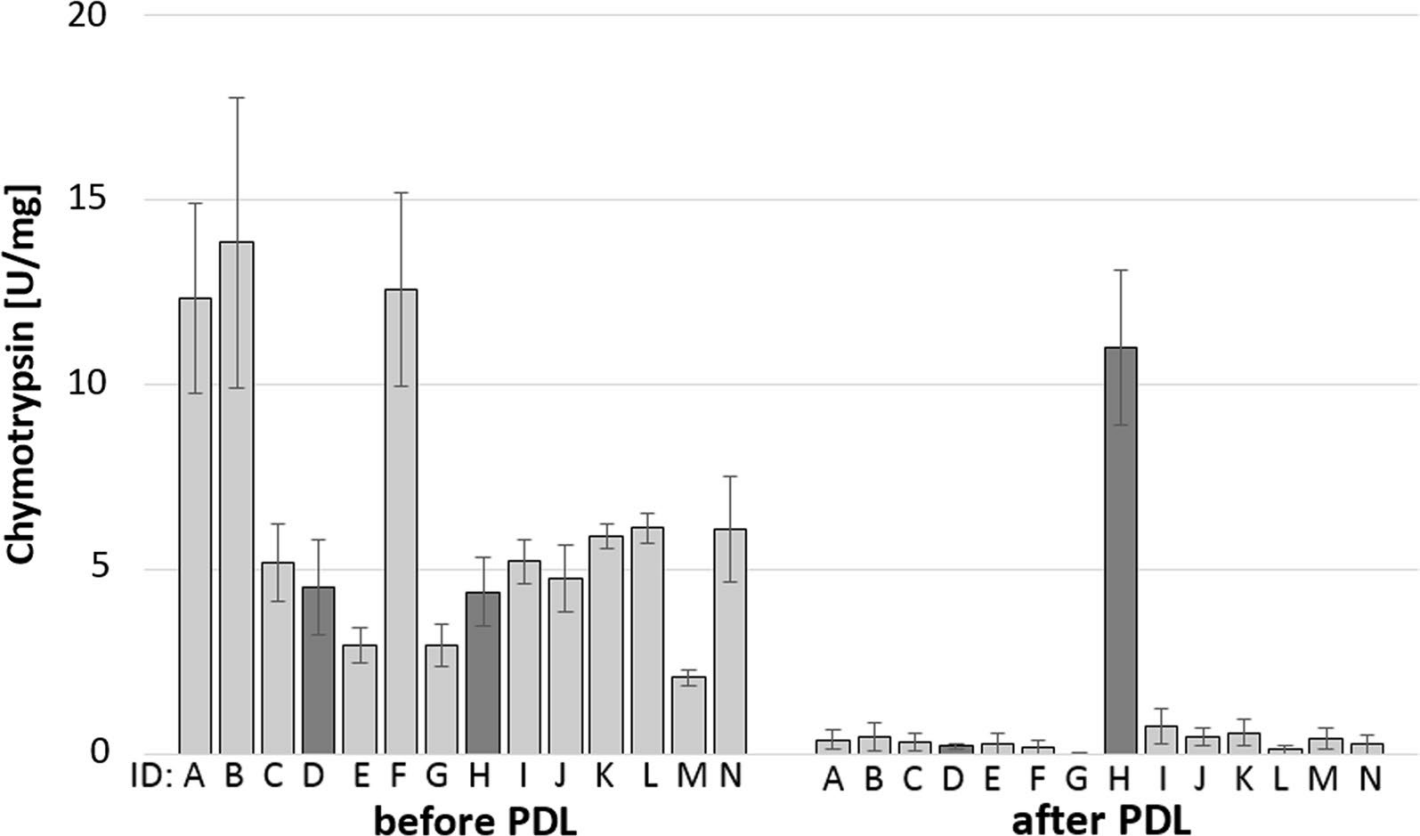
Fecal chymotrypsin content in minipigs before and six weeks after pancreatic duct ligation (PDL). Pigs were fed with a high-fat diet (18.5%) for seven days twice a day. Feces were collected on days 5–7, and chymotrypsin activity was then determined. Animal identification (ID) ranged from A to N. Dark columns mark the results of animal D with a decrease in chymotrypsin absorption and animal H with an increase after pancreatic duct ligation

Postoperatively, the mean fasting blood glucose level decreased significantly from 68.9 (± 23.5) mg/dl to 54.8 (± 8.1) mg/dl after pancreatic duct ligation (Table 1, p < 0.05). Feces turning clay colored while on the high-fat diet indicated exocrine pancreatic insufficiency. Fecal fat content increased significantly from 9.0% to 30.9% (Table 1, p < 0.001). The mean fat absorption decreased significantly from CFA 90.0 (± 2.8) % to CFA 24.2 (± 8.3) % after surgery (Table 1, p < 0.001). As shown in Fig. 3, two Goettingen minipigs (ID: D and H) had only minor changes in their CFA values before and after PDL. Animal D´s fat absorption remained almost constant (CFA: 91.3% to 90.6%) and H showed a significant but mild decrease (CFA: from 86.0% to 74.6%; p < 0.001). Due to the minor changes in fat absorption, these two minipigs were considered not exocrine insufficient and were therefore excluded from the study. Fecal pancreatic enzyme content was analyzed using chymotrypsin activity as a marker. Chymotrypsin activity in the Goettingen minipigs before surgery varied between 2.1 (± 0.2) U/g and 13.8 (± 3.9) U/g (Fig. 4). Postoperatively, chymotrypsin activity decreased significantly ranging from 0 (± 0.0) U/g to 0.33 (± 0.19) U/g in all animals (p < 0.001), except pig H who showed an increase from 4.4 (± 0.9) to 11.0 (± 2.1) U/g. Interestingly, animal D developed exocrine pancreatic insufficiency according to the chymotrypsin level, but showed no differences in fat digestion. All animals kept a stable weight six weeks after surgery while being fed a regular pig maintenance diet (Table 1). Additional enzyme substitution was not given in the period following PDL since all animals gained weight and showed no immediate adverse effects. All animal health care scores were within the normal range.

Animals D and H were sacrificed 89 and 76 weeks after surgery to investigate the reason for the maintained exocrine function despite pancreatic duct ligation. The pancreas of animal D was reduced to pigeon egg-size and was located next to the duodenum. In addition to this macroscopic finding, histologically a disseminated interstitial fibrosis of the organ was observed. However, a pancreatic duct was not detectable. Animal H had a normal pancreas, with the upper lobe measuring 10 cm and the lower lobe measuring 30 cm. The ligation had failed to occlude the pancreatic duct. The other animals were left alive for further studies. A few weeks after completed wound healing, the animals were brought together in small groups.

During the following four years, the animals were repeatedly used for PERT studies. The health status and body weight were monitored weekly. Blood glucose and vitamin levels were analyzed from blood samples taken under sedation.

**Fig. 5:**
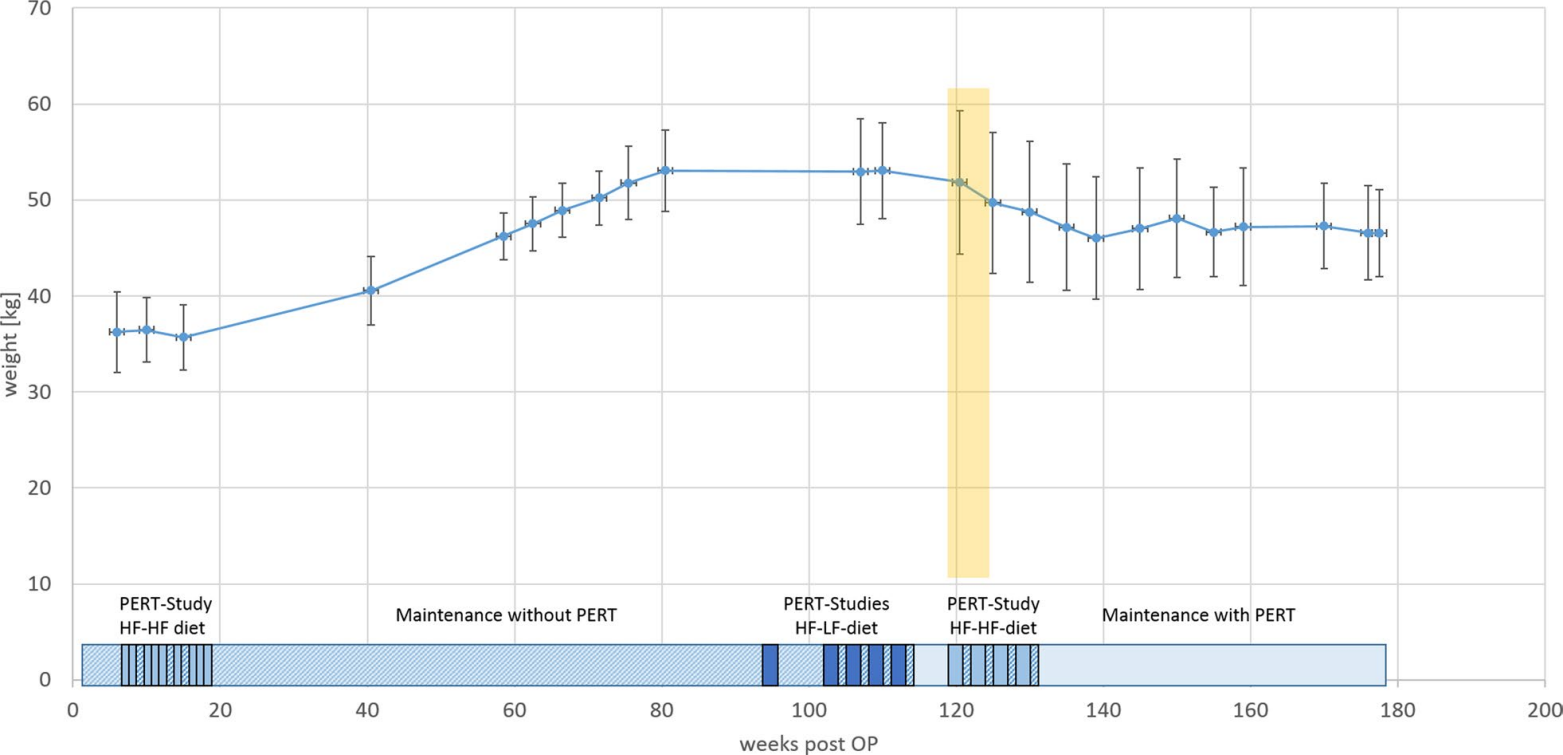
Body weight of pancreas duct ligated minipigs post-OP in long-term husbandry. Values are mean ± standard deviation. Yellow area marks change of maintenance fodder. Conducted PERT Studies are shown on a timeline below. PERT Studies with high fat-high fiber (HF-HF) diet are light blue, PERT Studies with high fat-high fiber (HF-LF) diet are dark blue, Maintenance phases without PERT are dashed. Animals have not been ligated on the same date. First PERT study was conducted for all animals exactly at 6–18 weeks post OP respectively. Further studies were conducted with animal groups with mixes ligation dates. Shown are average values of weeks post OP

In the phase following PDL, the animals developed normally and showed a continuous increase in weight up to 53 ± 5 kg (approx. up to week 80–100), indicating a low influence of PDL on food digestion and health state without digestive enzyme supplementation (Fig. 5).

To control weight increase, the former husbandry feed was switched to a lower-energy and fiber-richer feed. This initially led to a weight loss and subsequent weight stabilization of the animals at an average of 47 ± 6 kg approx. 160 weeks post-OP (Fig. 5, time of the feed change was week approx. 120, yellow bar).

Increased burden on animals was generally only observed during the digestive studies. However, stress levels above score 1 were rarely observed. As expected, we observed steatorreha, flatulence, abdominal pain, diarrhea, or occasionally constipation. Animals that completely refused food for one day during the trials were released from the test phase and recovered under normal diet. The weight of the animals during the studies was largely constant. In > 96% of the weight changes recorded during the studies, the burden of weight loss was less than 5%. Occasionally, weight reductions up to 7% were observed but animals restored after study end.

The importance of vitamin supplementation in elderly EPI minipigs was first shown by an animal, which developed an abnormal behavior regarding eating habits after approx. 105 weeks post-OP. The animal developed inappetence, partly denied single feedings up to several feedings a week. This was accompanied by weight loss. No signs of infection or parasite infestation were observed. The abdomen was permanently soft, and bowel movements were normal. The animal was first treated with digestive enzymes and omeprazole (20 mg/per feeding) from week 124 post-OP to week 129 post-OP for 6 weeks. Since no behavior change occurred a blood test was performed in week 127 post-OP and showed decreased leukocyte levels and slight hints for a systemic inflammation. Consequently, Dexamethason was administered to the animal on 6 consecutive days (week 131 post-OP). Again, no improvement could be observed. Finally, intramuscular injection of vitamin B12 in week 135 post-OP led to a rapid behavior change. At that time, the animals scoring was considerably increased up to 2 and the weight loss amounted approx. 30% in relation to the maximum weight reached by this animal and approx. 12% related to OP weight. To achieve a stable weight, the feed amount for this animal was increased to 300 g per meal. Up to date, normal feeding and weight were restored with consequent supplementation with digestive enzymes and vitamins. Blood parameters and physical conditions have recovered. Since a second animal also developed similar symptoms, another complete blood test including the vitamin status was performed for all remaining animals. A significant decrease of vitamin B12 and vitamin E was detected throughout the cohort. We started the complete supplementation of all pigs with pancreatic enzymes, vitamins and vitamin B12 injection approx. 120–137 weeks post-OP. No further occurrences of this kind were observed.

**Fig. 6:**
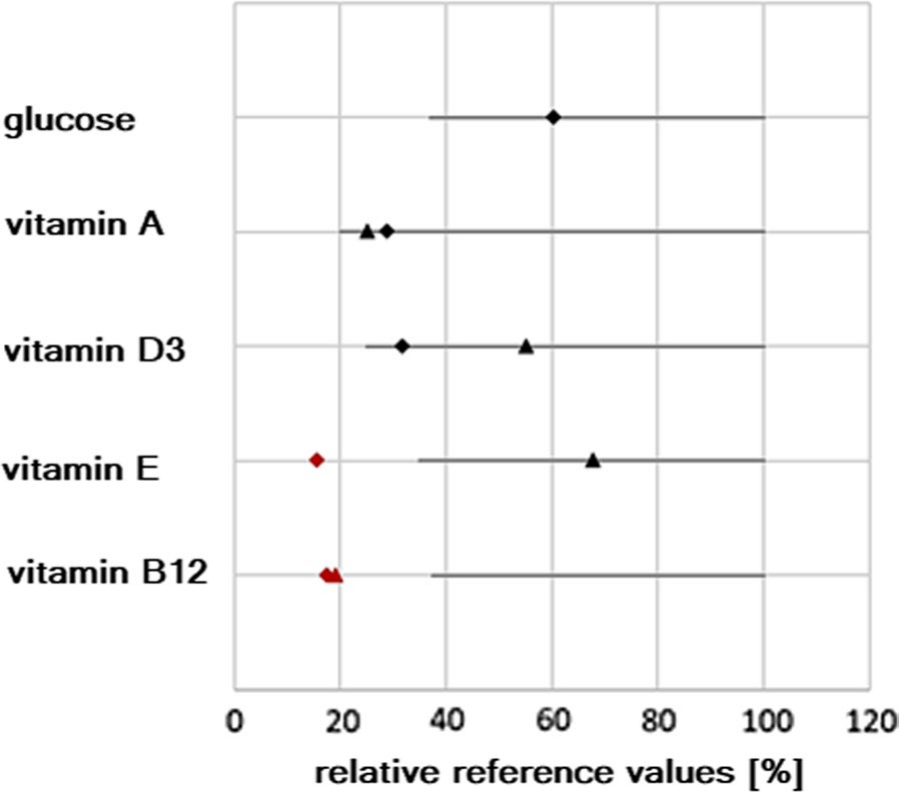
Blood level values of vitamins and glucose normalized to the reference values. The upper reference limit was set to 100%. Diamond = values before supplementation (130 ± 10 weeks post OP, n = 8). Triangle = values after supplementation (155 ± 9 weeks post OP, n = 5). Red color indicates values below reference range. All values are in percent and gray lines indicate reference range

To monitor the health status of the EPI pigs, blood chemistry was analyzed before and after treatment with pancreatic enzymes and supplementation of vitamins. The analysis of marker enzymes for acute pancreatitis (i.e. blood α-amylase and lipase, respectively) were not elevated approximately 60–80 weeks after surgery, so that no persisting pancreatitis was caused by PDL (data not shown). Before supplementation, the pancreatic duct ligated minipigs showed values within the reference range regarding blood glucose, vitamins A and D3. The ligation of the pancreatic duct led to a decrease below the reference range regarding vitamins E and B 12 (approx. up to week 125–134 post-OP, Fig. 6).

The supplementation with pancreatic enzymes and vitamins led to elevation of vitamins E and B12 and a level within the reference range was reached for vitamin E (Fig. 6) (approx. up to week 150–160 post-OP).

**Fig. 7:**
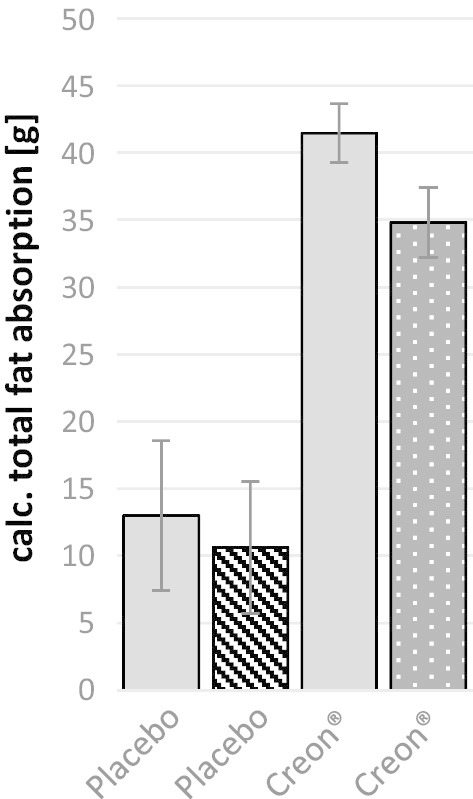
Total fat absorption based on CFA and theoretical dietary fat content in minipig six weeks (filled) after PDL and 97–126 weeks (dashed and dotted) after pancreatic duct ligation (PDL). Pigs underwent different PERT studies with three different dietary compositions (ca. 56 g fat (filled), 60 g fat high fibre (dashed), ca. 75 g fat low fibre (dotted)) and duration times. Pigs were fed twice a day over seven to fourteen days and feces was collected on days 5–7 or 10–14 and fat contend was determined. Mean values and standard deviations only from identical groups of animals were calculated to compare the results directly after the operation with those obtained about 2 years later. (Placebo n = 5; pancearin n = 5.). Statistical analysis was conducted using two-way analysis of variance (ANOVA) test with two-way, paired post hoc Tukey test. Whereas p < 0.01 = * slightly significant (≙Bonferoni korrection for n = 5)

The response to a PERT with the commercial pancreatin formulation was examined at different time points and ages of the animals. The studies differed in the food composition and slightly in the pancreatin dose. With the help of the determined CFA and the fat content of the feed combination, a normalization of the values to the total amount of fat absorbed was possible. Based on these studies, the change in fat absorption over 2 years could be compared without and with PERT (very similar pancreatic lipase dose, Fig. 7). There were no significant differences in the calculated total fat absorption of the respective animal groups. The response did also not depend on the used high fat diet. The total amount of fat absorption is very similar even after more than 2 years both with (pancreatin 41.5 ± 2.2 g or 34.8 ± 2.6 g) and without supplementation (placebo 13.0 ± 5.6 g or 10.6 ± 4.9 g).

## Discussion

This study describes for the first time in detail the perioperative management, surgical approach, and postoperative changes of pancreatic duct ligation in non-fistulated minipigs. This method was established for the induction of exocrine pancreatic insufficiency [9, 17, 18, 31, 32]. It is well-suited for experimental studies of pancreatic enzyme replacement therapies due to a similar physiology and anatomy of the gastrointestinal tract of humans and pigs [1]. Furthermore, both species are omnivorous, and a diet composed of comparable components can be used [33]. However, to date, a detailed description of the technical approach is lacking in the literature.

In contrast to humans, fulminant necrotizing pancreatitis does not typically occur after PDL in pigs. In several studies, a short phase of acute pancreatitis after PDL in pigs was observed, which was characterized by increased amylase and lipase activity in the blood. Nevertheless, these marker enzymes had returned to normal levels 25 days after surgery. Furthermore, no other symptoms of persistent pancreatitis were present [17, 34]. Our findings are in good accordance with the aforementioned studies as no persisting pancreatitis could be detected in our animals and normal behavior, physical condition and feed intake were restored one week postoperatively. However, histological signs of chronic fibrosing pancreatitis have been identified in pig necropsy studies [17, 32]. Maybe the separate drainage of the bile duct and the pancreatic duct generally found in pigs prevents a severe postoperative course [35]. Furthermore, the absence of pancreatic diseases as they occur in EPI affected humans (i.e. cystic fibrosis, acute or chronic pancreatitis) allows a testing of the advanced treatment strategies for EPI, which are not affected by parallel inflammatory processes.

Specific knowledge of the porcine abdominal topography is crucial for the surgery. A short extra parenchymal segment of the pancreatic duct forming a bridge between the duodenum and the pancreatic head can be found in most pigs [17]. It is embedded in a membrane and can be dissected and ligated easily. Preparation of the pancreatic blood vessels is not necessary to induce pancreatic insufficiency. Thus, pancreatic duct ligation does not significantly affect the endocrine function of the pancreas [32]. Several studies using histopathology and measurement of blood glucose or glucose tolerance tests have shown that even after several weeks of PDL the Langerhans islets showed no morphological changes and the insulin production was nearly unaffected [17, 36].

Postoperative changes of fecal fat content and altered chymotrypsin levels are useful indicators of successful induction of pancreatic exocrine insufficiency in pigs as seen in other studies and confirmed by our data [9, 17, 37]. Blood glucose levels were initially significantly reduced after surgery but did neither reach hypoglycemic levels nor cause any symptoms. This might be caused by postoperatively altered beta cell functions [15]. In contrast to humans with chronic pancreatitis, pigs do not develop severe acute or chronic pancreatitis or diabetes and generally recover [1, 22]. In our case even 125—134 weeks after surgery, none of the animals developed diabetes (Fig. 6). This is in good accordance with the aforementioned studies in which the endocrine function of the pancreas was maintained even after several weeks after PDL [17, 36]. Furthermore, glucose clearance was investigated in EPI animals with and without PERT and an improvement was found under PERT [36]. The authors therefore suggest acino-insular communication, which regulates the peripheral glucose utilization. In this regard, the development of diabetes in our study animals is unlikely since the animals are supplemented with pancreatic enzymes.

As shown in this study, reduced fat absorption and fecal chymotrypsin levels indicated induction of exocrine pancreatic insufficiency in 12 out of 14 animals after pancreatic duct ligation. This allows the investigation of a broad range of scientific topics and supports future experimental studies. 12 pigs with a low CFA showed low chymotrypsin concentrations as expected. However, two animals did not develop exocrine pancreatic insufficiency regarding fat absorption; chymotrypsin levels were inconclusive. In animal H, the pancreatic duct ligation had failed, and in animal D, the reason for the presence of a subtotal atrophic pancreas remained unclear. The atrophic pancreatic tissue of D obviously led to an impaired and low chymotrypsin level. The observed atrophy as well as fibrosis of the organ are in good agreement with the observations from other studies in which pancreatic duct ligation was performed in pigs [17, 36]. A reduction in pancreatic azini associated with fibrosis has been observed on several occasions. At the same time, the morphological as well as functional preservation of the insulin-producing Langerhans islets could be confirmed. However, the CFA was normal after surgery. Most likely, enough enzymes could reach the intestine, preserving sufficient digestion through an accessory pancreatic duct. Such an accessory pancreatic duct can be found in 10% of pigs [18, 38]. However, in our study, none could be identified for ligation. Therefore, a thorough examination of the membrane between the duodenum and the pancreatic head is necessary, as is the precise ligation of the pancreatic duct (Figs. 1a, 2a).

In one pig, the chymotrypsin activity level failed as a marker for detecting exocrine pancreatic insufficiency regarding insufficient fat absorption. Although this might be a single case, it suggests that the CFA should also be considered in further studies.

This model easily allows sufficient determination of CFA values from excreted feces to successfully analyze lipases with various dosages or different enzyme formulations in enzyme replacement therapy. The investigation of additional parameters such as nitrogen or carbohydrates to study supplemented protease and amylase activity requires modification of the study design [7]. Here it is crucial to analyze the chyme after passage through the small intestine [1]. In contrast to the ileo-cecal re-entrant fistulated EPI model the here described non-fistulated animals can be held under normal housing conditions without greater care. This results in a robust model with improved animal health. The major benefit is the possible long-term use of animals with a good health state within PERT studies.

The possibility of keeping the animals in small groups, if no digestive studies were conducted, is consistent with the social structure of pigs, for whom individual husbandry is considered an increased burden. Furthermore, the preference for female animals is recommended, as they show a high social compatibility even when the husbandry alternates between individual and group housing. In contrast, fistulated animals must be kept permanently in individual boxes, as otherwise the risk of injury to and around the fistula by other animals is high. Moreover, the boxes must be specially equipped, so that the animals cannot scratch the fistula at parts of the boxes. The social contact to the other animals is restricted. The fistulas must always be cleaned several times a day. Furthermore, the animals can only eat very fine, liquid food. Therefore, the fistulated animals need permanent and intensive care by specially trained animal keepers and veterinarians. Moreover, during the chyme collections, the animals must be kept for a few days in special metabolic boxes.

In contrast, the non-fistulated EPI model allows keeping the animals during study pauses on straw. This has two positive effects on the pig. First, it can contribute to its recovery from high fat diet and health status through "straw-eating". Second, at the same time straw is an important part of the enrichment. The straw is very important for the animals’ well-being as it stimulates the instinct of pigs, they can chew on it, rummage through with the snout or build sleeping places and keep themselves clean. This type of enrichment enables the animals to demonstrate their natural behavioral repertoire and ensures that the animals are in a very good physical and mental condition in the long term.

Another important factor for long-term usage of this EPI model are resting phases of at least 6 weeks between the experimental series. This period of time is needed for the animals to recover from the burden of the high-fat diet and to restore a physiological state of the digestive tract.

A further advantage of the non-fistulated model is that animals’ diet is not restricted to liquid food. Whereas in the fistulated EPI model only low raw fiber content of the feed is used and the feed must be very finely ground to prevent complications with the passage of the chyme through the cannula [39]. Ingesting more solid food not only brings the model closer to the situation of human patients, but also allows the use of non-homogeneously mixed study diets, as expected in human foods.

An increase in the burden on the animals generally only occurred during feeding studies. Feeding high fat contents and medication could cause fatty stools, flatulence, abdominal pain, diarrhea, or constipation. It is particularly important for the animal welfare and the stability and quality of the study results to design the order of the studies carefully. In order not to jeopardize the enduring health of the animals, care must be taken during longer-lasting test phases that high-fat diets in combination with no or little effective medication are followed by effective medication or study breaks.

However, although most of the animals developed normally referred to weight gain and general conditions, one minipig developed an abnormal behavior over the time, that resulted in lack of appetite and severe weight lost. It is known in human EPI patients, that the functional loss of the exocrine pancreas causes a lower pH in the intestine which in turn diminishes the activity of the few residual gastric enzymes [1]. Keeping this in mind, we choose the administration of pancreatic enzymes (commercial pancreatin) and proton pump inhibitor (Omeprazol) as our therapeutic approach. The application of this medicine for 6 weeks had no influence on the eating behavior. The analysis of the blood count revealed a reduced level of leukocytes (data not shown) and a mild inflammation was assumed. For this reason, a glucocorticoid (Dexamethason) was administered to the animal. Again, no influence on the eating habit could be observed. Since another known comorbidity occurring in EPI patients is a malabsorption of fat- soluble vitamins and vitamin B12 [26, 40], our next therapeutic approach was an intramuscular injection of vitamin B12. Fortunately, the animal immediately changed its eating behavior and ate regularly which led to a slow but steady increase in weight. Another animal displayed a similar behavior but with significantly less refusal to feed and therefore less weight loss approx. 109 weeks post-OP. The successful vitamin B12 therapy was also chosen for the second pig and had the same positive effect. A following renewed blood test on all remaining animals revealed a vitamin deficiency especially of vitamin B12 as well as the fat-soluble vitamin E.

Especially the absorption of vitamin B12 is dependent on a secretion of the pancreatic juice.

Proteins play an important role in the uptake of vitamin B12. In the diet, vitamin B12 is initially strongly bound to proteins. Responsible for the absorption, however, is the intrinsic factor (IF), which has a significantly lower affinity to bind vitamin B12 than the other proteins. By the presence of pancreatic enzymes, these other proteins are desirably degraded under basic conditions so that the balance shifts in favor of the vitamin B12-IF complex, which can be absorbed [41, 42]. The fact that a vitamin depletion of the animals was detected only at a late stage in elderly animals, was probably due to the high depot effect of vitamin B12 in the liver [43].

With the immediate supplementation of the animals, especially with vitamin B12, no further occurrences of this kind were observed. Monitoring the EPI minipigs showed that blood vitamin and glucose concentrations of the test group animals were very similar. Accordingly, it is not necessary to regularly withdraw blood in EPI animals with normal behavior and weight. For blood value monitoring as few as possible blood samples are taken mostly from different animals. This is done to keep the burden on the animals low and still keep an overview of the progress of the blood parameters of the whole group.

Through comparative studies immediately after PDL and around 2 years later, we were able to show that the CFA in the animals hardly changed over this period. Regardless of the composition of the high-fat diet, the animals showed no significant differences in the absorption of fat. Both immediately after PDL and about 2 years later, the fat absorption during placebo control was, as expected, low and strongly increased under PERT. Accordingly, this model can also be used over a very long period and the animals can be used as their respective controls. A repeated determination of data on particularly stressful placebo controls is therefore not necessary.

PERT studies can be done at any time with the described EPI minipigs. Before a new experimental series, a washout phase of one week with no enzyme supplementation was maintained. This is necessary to avoid influencing the following study by the given additives. Vitamin supplementation is restricted to washout phases as well.

Our data demonstrate that a carefully performed surgical procedure creates a valuable EPI minipig model without severe side effects for the treated animals. No signs of diabetes occurred, all health scores were normal, and, most importantly, the animals’ weight remained constant with a normal diet without a pancreatic enzyme replacement therapy for about 2 years. However, if this model is to be carried out in long-term studies beyond this period, it is strongly recommended to supplement the animals with pancreatic enzymes and vitamins right from the start to avoid fat soluble vitamin dependent weight loss. Taken this into account the non-fistulated model provides the potential for the long-term study of EPI therapies in Goettingen minipigs.

## Conclusions

Pancreatic duct ligation is an excellent technique to induce exocrine pancreatic insufficiency in Goettingen minipigs. The EPI model can be quantitatively assessed and is well-tolerated by the animals. If animals are supplemented accordingly, they can be kept in good general health status with age independent and reproducible PERT studies for many years. This simplified, non-fistulated minipig model provides access to the testing of advanced treatment strategies for EPI, as the conditions for performing PERT studies have been significantly relieved and further developed, particularly in terms of the 3R principle.

## Data Availability

The datasets used and analysed during this study are available from the corresponding author on reasonable request.

## Abbreviations

CFA: Coefficient of fat absorption
EPI: Exocrine pancreatic insufficiency
PDL: Pancreatic duct ligation
